# Selected Aspects of the Intricate Background of Immune-Related Cholangiopathies—A Critical Overview

**DOI:** 10.3390/nu15030760

**Published:** 2023-02-02

**Authors:** Beata Kasztelan-Szczerbinska, Anna Rycyk-Bojarzynska, Agnieszka Szczerbinska, Halina Cichoz-Lach

**Affiliations:** 1 Department of Gastroenterology with Endoscopy Unit, Medical University of Lublin, 8 Jaczewski Street, 20-954 Lublin, Poland; 2 Medical University of Warsaw, 61 Zwirki i Wigury Street, 02-091 Warsaw, Poland

**Keywords:** primary biliary cholangitis (PBC), primary sclerosing cholangitis (PSC), bile acids (BAs), gut microbiome (GM), ursodeoxycholic acid (UDCA), obeticholic acid (OCA), agonist of farnesoid X receptor (FXR)

## Abstract

Primary biliary cholangitis (PBC), and primary sclerosing cholangitis (PSC) are rare immune-related cholangiopathies with still poorly explained pathogenesis. Although triggers of chronic inflammation with subsequent fibrosis that affect cholangiocytes leading to obliteration of bile ducts and conversion to liver cirrhosis are unclear, both disorders are regarded to be multifactorial. Different factors can contribute to the development of hepatocellular injury in the course of progressive cholestasis, including (1) body accumulation of bile acids and their toxicity, (2) decreased food intake and nutrient absorption, (3) gut microbiota transformation, and (4) reorganized host metabolism. Growing evidence suggests that intestinal microbiome composition not only can be altered by liver dysfunction, but in turn, it actively impacts hepatic conditions. In this review, we highlight the role of key factors such as the gut–liver axis, intestinal barrier integrity, bile acid synthesis and circulation, and microbiome composition, which seem to be strongly related to PBC and PSC outcome. Emerging treatments and future therapeutic strategies are also presented.

## 1. Introduction

**Figure 1:**
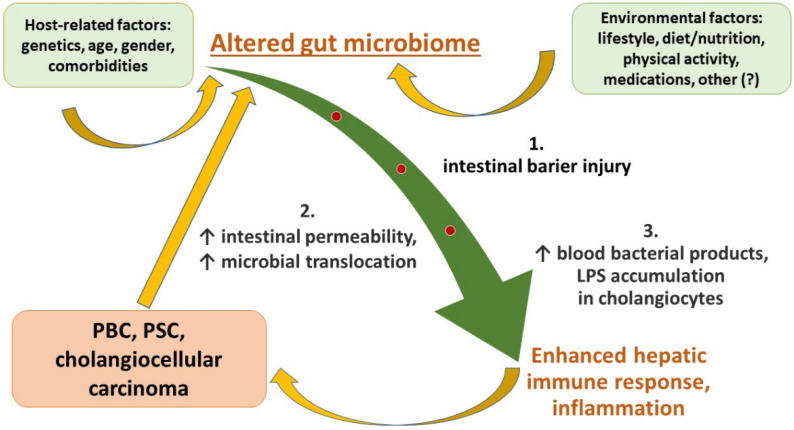
The vicious circle of gut–liver crosstalk in the course of immune-related cholangiopathies.

Primary biliary cholangitis (PBC) and primary sclerosing cholangitis (PSC) are progressive immune-related cholangiopathies. They are regarded as orphan disorders, but increasing PSC incidence and prevalence in Europe, and rising PBC prevalence across Europe, North America, and the Asia-Pacific region have been reported recently. Prevalence per 100,000 for PBC ranges from 1.91 to 40.2, and for PSC between 0.78 and 31.7 [1]. The etiologies of PBC and PSC are unclear and seem to involve a combination of genetic and environmental factors. Their clinical signs and symptoms are related to cholestasis and its direct health consequences. Progressive destruction of the bile tree due to extensive inflammation and fibrosis with altered bile flow through intra- or extrahepatic bile ducts and secondary hepatocellular damage that occur in the course of both immune-related cholangiopathies lead to retention of excessive bile acids, hydrophobic bile salts, and damaging metabolites that under normal conditions are excreted into the bile. Their body accumulation results in injury to cell membranes and liver cells are mostly affected. Although the main effects of cholestasis concern the liver and gut function, secondary ones may affect every host organ. Cholestasis seems to play a critical role in the modulation of gut homeostasis and patient nutritional status, which is considered a relevant and prognostic factor with a significant impact on the effects of management and treatment of patients with liver disorders of different etiology. Also, patient nutritional status, in turn, may influence liver metabolic functions. As a modifiable condition, it should be assessed early enough in the course of liver disease to introduce appropriate medical interventions and prevent further host complications that may include infections, hepatic encephalopathy, ascites, and lower survival [2], as well as improve patient quality of life. Recent lines of evidence indicate that gut microbiome composition can be altered by liver dysfunction, but also in turn affects host hepatic conditions. Therefore, the role of bile acids (BAs) and gut microbiota interactions have been extensively studied. The recognition of multiple-step defective mechanisms involved in the complex pathogenesis of the aforementioned cholangiopathies has led to an improved understanding of the underlying pathways of human cholestasis. Herein, we provide a comprehensive summary of the potential role of various factors, such as the gut–liver axis, intestinal barrier integrity, BA synthesis and circulation, and microbiome composition, which seem to be strongly associated with PBC and PSC outcomes. Since the etiopathogenesis of both disorders remains poorly understood, therapeutic options are limited and not satisfactory [3,4,5]. Our review looks into recent evidence elucidating the role of various factors involved in PBC and PSC development and discusses their potential therapeutic implications. We hope that increased awareness of the complicated mechanisms engaged in the course of immune-related cholangiopathies might improve patient medical management and subsequently decrease the need for liver transplantation. The intricate background of both immune-related cholangiopathies described further in our review is presented in Figure 1.

## 2. Clinical Characteristics of PBC and PSC

Immune-related cholestatic liver diseases including primary biliary cholangitis (PBC) and primary sclerosing cholangitis (PSC) manifest as an impairment or disruption of bile production and accumulation of toxic bile acids and metabolites. The majority of PBC and PSC patients present with typical disease signs and symptoms and do not require a liver biopsy. PBC and PSC left untreated lead to advanced liver fibrosis and subsequent liver failure [6].

PBC is an uncommon, chronic cholestatic disorder related to the autoimmune granulomatous destruction of small intrahepatic bile ducts. The condition is progressive in most patients; however, some PBC patients may show no disease symptoms. The number of asymptomatic individuals is increasing due to the widespread use of both liver function tests and antimitochondrial antibody assays [7,8]. The remaining patients may present with fluctuating elevation of cholestatic enzymes such as ALP and GGT and signs of chronic liver disease. Quality of life in PBC patients can be severely altered by pruritus, fatigue, and Sjögren’s syndrome, i.e., sicca syndrome that may be persistent and intense even in the early disease stage. Noteworthily, symptoms do not correlate with the disease course severity, but are related to its duration, About 40% of PBC patients suffer from skin complaints that include not only dry skin but also jaundice, hyperpigmentation, xanthelasma, xanthomas, and dermatographism [9,10]. Metabolic bone disease in PBC patients can develop as a result of reduced absorption of fat-soluble vitamins, i.e., vitamin D deficiency, and therefore they should be monitored and treated if required. However, the development of liver cirrhosis with its complications finally determines the disease prognosis [11].

PBC develops as a result of decreased expression of the Cl−/HCO3− anion exchanger AE2 in the biliary epithelium in patients with a genetic predisposition to autoimmunity. Epigenetic factors such as miR-506 upregulation and methylation of AE2 promoter are considered the cause of AE2 downregulation [12]. Cholangiocyte damage occurs due to AE2-related decreased secretion of biliary bicarbonates and subsequent so-called alkaline umbrella disturbance with increased cellular penetration of apolar toxic bile acids [13]. Moreover, increased intracellular pH in the biliary epithelium induces the activation of adenylyl cyclase, resulting in further bile salt-associated cholangiocyte apoptosis. It was proposed recently that the AE2 defect may also disrupt cholangiocyte mitophagy, leading to mitochondrial dysfunction and oxidative stress, as well as host immune cell exposure to mitochondrial antigens with anti-mitochondrial antibody (AMA) production [3,14]. AE2 downregulation is also present in immune cells, particularly in CD8+ T cells, and may cause autoimmune T-cell reactions. The loss of tolerance towards self-antigens induces cellular and humoral immune responses towards cholangiocytes, although why in PBC only small intrahepatic biliary epithelial cells are targeted while mitochondria are present in all cells remains obscure [15]. Anti-mitochondrial antibodies (AMAs) directed against the E2 subunits of the 2-oxo acid dehydrogenase complexes (PDC-E2) represent the hallmark of PBC and can be confirmed in 90–95% of patients [16]. The discovery and cloning of the AMA target antigen in 1987 started the progress in the understanding of PBC [17]. Notably, the presence of AMAs in the blood is not sufficient to diagnose PBC, because they can be also detected in less than 1% of healthy subjects [16]. AMAs may be also present in patients with overlap syndromes and other liver diseases (i.e., nonalcoholic steatohepatitis, viral hepatitis), as well as with inflammatory myositis and heart disease [16]. Furthermore, AMA titers do not correlate with disease activity and severity, so they should not be serially followed [18]. The European Association for the Study of the Liver (EASL) recommends testing for PBC-specific antinuclear antibodies (ANAs) such as anti-sp100 and anti-glycoprotein 210 (anti-gp210) antibodies, especially in patients who are AMA-negative, but who present with cholestasis [19]. Most patients with PBC do not require a liver biopsy to establish the diagnosis. A biopsy is essential especially when an overlap syndrome is suspected, e.g., PBC/autoimmune hepatitis (AIH). Although the term “overlap syndrome” has been historically used, most of the American College of Gastroenterology (ACG) experts favor the term “PBC with autoimmune features” [18]. Ninety-five percent of patients with PBC are women. It can also occur in children [20]. According to data reported by EASL, the estimated PBC incidence is 1 per 1000 women over the age of 40. The female predominance remains unclear, as in many autoimmune diseases [19]. Retrospective analysis indicated that the female:male ratio was 9:1 in the early 2000s. [21]. However, currently, growth is observed in male incidence [19,22]. Nowadays, the female:male ratio is estimated to be 5:1 or even 2.1:1 in most recent studies [22,23]. Lleo et al. reported higher overall mortality for males than females among PBC patients (10-year survival rates of 67% for females and 47% for males) [22]. PBC is associated with a strong genetic predisposition among identical twins [24]. The disease should be suspected in all individuals presenting with elevated alkaline phosphatase (ALP) and gamma-glutamyl transpeptidase (GGT) and high levels of conjugated bilirubin, in coexistence with pruritus and/or fatigue, and/or dilated bile ducts.

The term “cholangitis” has replaced the term “cirrhosis” in the acronym “PBC” to adequately describe the histological hallmark of dense inflammatory infiltrates around damaged intralobular bile ducts [18]. As indicated by Beuers et al. [25], the changed terminology helped PBC patients to lose the stigma of cirrhosis, poor prognosis, and alcohol-related implications, as well as improve their professional and social lives.

If left untreated, PBC can lead to liver cirrhosis, which increases the risk of hepatocellular carcinoma (HCC) [23]. Predictors of poorer PBC outcome include male sex, younger age, i.e., <45 at disease onset and advanced disease at presentation [9]. Biochemistry tests are used for patient prognosis and treatment response monitoring.

Primary sclerosing cholangitis (PSC) also represents a rare cholestatic hepatobiliary disease characterized by immune-mediated chronic inflammation that slowly damages intra- and extrahepatic bile ducts leading to multifocal bile duct strictures and progressive liver fibrosis [26,27]. The condition may finish as biliary cirrhosis with portal hypertension and end-stage liver failure. PSC is more commonly diagnosed in men between the ages of 30 and 40. Many PSC patients do not present any clinical disease signs or symptoms at the time of diagnosis [28]. Diagnosis of cholestasis with increased bilirubin, serum alkaline phosphatase (ALP), and gamma-glutamyl transferase (GGT) values, can be established during routine health evaluation or high-risk patient screening, i.e., patients with inflammatory bowel disease (IBD). Nevertheless, 30% to 40% of patients present with normal ALP at diagnosis or during their disease course, and the majority of them have normal serum total bilirubin levels at the time of diagnosis [28]. In those who present with symptoms, abdominal pain is observed as the most frequent symptom (20%), followed by pruritus (10%), jaundice (6%), and fatigue (6%) [29]. Liver and spleen enlargement can be observed in 44% and 39% of patients, respectively [30]. PSC is strongly associated with inflammatory bowel disease (IBD), including ulcerative colitis (UC) and Crohn’s disease (CD), with the prevalence ranging from 67% to 73% [31]. Ulcerative pancolitis is observed in 94% of PSC-UC and colitis in 96% of PSC-CD patients [32]. PSC increases the risk of multiple gastrointestinal (GI) tract neoplasia such as hepatocellular carcinoma (HCC), cholangiocarcinoma (CCA), colorectal (CRC), and pancreatic (PC) cancers [33]. Although no serological hallmarks have been identified for PSC, IgG and ANA are frequently elevated, and high IgG4 levels may signal the presence of a unique IgG4-related PSC variant. Since numerous patients with PSC present with no signs or symptoms, the disease is frequently detected through altered results on routine liver blood tests. The final PSC diagnosis is usually established based on cholangiography. Characteristic imaging findings of thickened, inflamed bile ducts, dilatation of intra- and extrahepatic ducts, and focal strictures can be seen on magnetic resonance (MR) cholangiography, a first-choice noninvasive diagnostic alternative to endoscopic retrograde cholangiopancreatography (ERCP) [34]. PSC targets mainly medium to large intra- and extrahepatic bile ducts [35,36]. The course of PSC can be complicated by bacterial cholangitis, gallbladder polyps, and, as mentioned previously, cancer development. CCA remains the most common malignancy in patients with PSC with a cumulative lifetime risk of about 10–20%. It accounts for a large proportion of PBC mortality. There is no proven medical or interventional therapy in PSC so far [27]. Liver transplantation (LT) remains the only life-extending therapeutic approach for eligible patients with end-stage disease and is ultimately required in approximately 40% of patients, typically about 10 years after being diagnosed with PSC [28]. Nevertheless, as reported in a recent international multicenter study, LT for PSC may be complicated by illness recurrence (rPSC) in up to 25% of recipients, and inflammatory conditions both before and after LT play a critical role in rPSC incidence [37].

## 3. Genetic and Epigenetic Determinants in Susceptibility to PBC and PSC

As already mentioned, the etiology of PBC is unknown, but an individual’s disease susceptibility is thought to be influenced by a combination of genetic predisposition and environmental factors [15,17]. Several genome-wide association studies (GWAS) and Immunochip studies reported a nearly consistent genetic susceptibility among populations from different European areas, with a significant association of more than 20 loci with PBC [24,38,39]. The autoimmune background of PBC is indicated by the location of PBC-related single nucleotide polymorphisms (SNPs) in genes encoding molecules of the immune system (i.e., Human Leukocyte Antigens (HLA) class II, Il-12A, and Signal Transducer And Activator Of Transcription 4 (STAT4)) [39,40]. A recent Chinese study confirmed previously described PBC risk loci, as well as identified novel alleles such as IL21, IL21R, CD28/CTLA4/ICOS, CD58, ARID3A, and IL16, supporting the critical role of the IL-21 signaling pathway in disease development [24]. However, most risk variants have no significant effect on disease susceptibility. Among PBC patients, concordance rates in monozygotic twins are estimated to be 63%, while in other autoimmune diseases (AIDs) it is less than 50% [39,40]. Unfortunately, there are still many loci that were not identified with GWAS because they are not statistically significant. Furthermore, among many identified loci, most of them do not have a meaningful effect that would explain the possible etiology of PBC [39].

Growing evidence indicates the involvement of epigenetics in PBC and PSC pathogenesis [38,39]. Epigenetics relies on transcriptional modifications with no change in the nucleotide sequence. It may form a bridge between genetic predisposition and environmental factors and be relevant to disease development and evolution. Epigenetic modifications that have already been demonstrated in PBC include methylation of DNA, posttranslational modifications of histone proteins, and noncoding RNAs that may lead to altered gene expression [38]. As mentioned before, miR-506 upregulation and methylation of the AE2 promoter have a critical role in PBC etiopathogenesis [12]. Moreover, changes in the DNA methylation pattern could account for the differences found in the concordance rate between monozygotic twins with PBC [3].

The genetic involvement in PSC pathogenesis has been also established. Twenty-two susceptibility loci for PSC have been established by GWAS, with the human leukocyte antigen (HLA) complex presenting the strongest finding [41,42]. Available PSC genetic data explain less than 10% of disease facts, and environmental risk factors probably account for more than 50% of the indeterminate part [41,42]. The association of fucosyltransferase 2 (FUT2) gene polymorphisms with PSC, Crohn’s disease, and biochemical features of cholestasis has been found. FUT2 and HLA genes may represent a valid link between epigenetic and environmental factors [40,41]. Notably, Maroni et al. investigated the role of FUT2 in an animal model and reported that FUT2-/- mice presented with hepatic periductal fibrosis, microcirculatory disturbances of the liver, and sensitivity toward hydrophobic bile salt feeding [43].

As indicated elsewhere, genetic susceptibility to autoimmune liver diseases is based mainly on polymorphisms of genes encoding for the human leukocyte antigens (HLA). However, since 80–90% of PBC patients do not carry the most common HLA susceptibility alleles, non-HLA loci are suggested to contribute to disease development [44,45]. Recent worldwide GWAS analyses revealed more than 40 non-HLA alleles likely related to PBC [23]. With regard to PSC, several HLA and non-HLA risk loci have been identified, although their role appears to be negligible in contrast to environmental determinants [46]. Since significant variation in PSC distribution between geographically separated populations has been reported, the above data may also indicate the importance of diverse environmental exposure [40]. Nevertheless, the PSC risk among first-degree relatives is estimated as more than 80 times higher, which shows the relevance of inherited disease characteristics [47]. Elucidation of the genetic background opens chances for a better understanding of the etiopathogenesis of both cholangiopathies, and as a result, developing more effective therapy.

## 4. Bile Acids and Their Importance in the Liver and Gut Metabolism

Despite the immune-related pathomechanisms confirmed in PBC and PSC, their response to immunosuppressive treatment is poor. Therefore, choleretic treatment with ursodeoxycholic acid (UDCA), which improves PBC, but not PSC prognosis, is used as a first-line therapy [11,28]. The aforementioned data indicate the critical importance of bile composition in disease etiopathogenesis. As mentioned earlier, AE2-related decreased biliary bicarbonate secretion and alkaline umbrella disturbance promote cellular penetration of apolar toxic bile acids (BAs) and further cholangiocyte injury [13].

There is growing evidence that the liver can communicate and regulate gut function by releasing BAs and immune-related mediators. BAs exert numerous physiological effects on the host metabolism by interaction with intestinal microflora. Recent reports suggest that profiles of BAs may change considerably with distinct etiology of liver disease [48,49,50,51,52]. Bile acids represent heterogeneous amphipathic molecules that have both polar (water-soluble or hydrophilic) and apolar (water-insoluble or hydrophobic) parts, and therefore they can dissolve in water as well as in fat [53]. The regulation of cholesterol homeostasis remains their main role in the host. The better water solubility in comparison with their precursor promotes cholesterol removal from the human body [48,53]. The proper bile ratio of BAs and cholesterol prevents cholesterol precipitation and further formation of gallstones; therefore, the loss of BAs increases the risk of cholesterol stone development [53,54].

### 4.1. Bile Acid Synthesis and Circulation

Bile acids are synthesized in the human liver through the cholesterol oxidation process mediated by cytochrome P450. Seventeen enzymes are involved in their biosynthesis and about 500 mg of cholesterol is biotransformed to BAs within 24 h in adults [55,56]. There are four main BAs in human bile:

1. primary BAs, which are synthesized in the liver and secreted with bile into the small intestine: cholic acid (CA) and chenodeoxycholic acid (CDCA);
2. secondary BAs, which are formed from primary BAs in the large intestine under the influence of bacterial enzymes: deoxycholic acid (DCA) originating from cholic acid, and lithocholic acid (LCA) originating from chenodeoxycholic acid;
3. tertiary BAs, which are metabolites of major BAs including ursodeoxycholic acid (UDCA), which is currently used to treat liver diseases and bile duct disorders [53].

**Figure 2:**
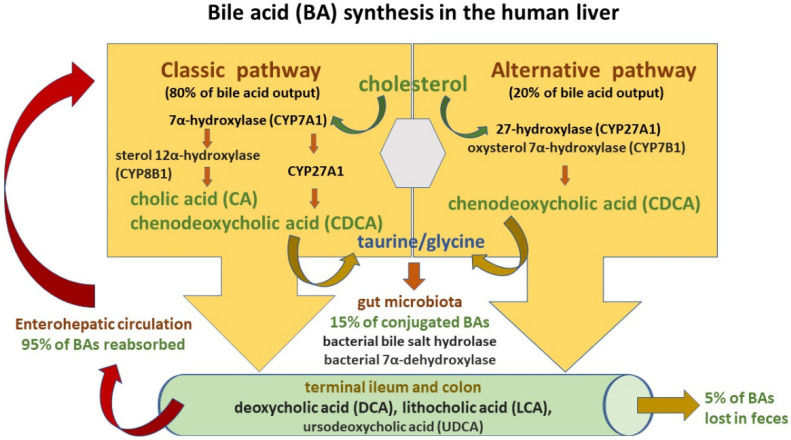
Bile acid (BA) synthesis and circulation.

The BA synthesis and circulation are presented in Figure 2.

Two pathways are engaged in this process: the classic and alternative [48,50]. The classic pathway of BA synthesis accounts for about 75% of BA output and is catalyzed by cholesterol 7α-hydroxylase (CYP7A1), which via a rate-limiting manner produces the primary cholic acid (CA) and chenodeoxycholic acid (CDCA) [49,53]. On the other hand, the alternative pathway is associated with the activity of mitochondrial sterol 27-hydroxylase (CYP27A1) and results in CDCA output. Primary BAs are then conjugated predominantly with glycine and to a lesser extent with taurine (at a ratio of 3:1) before their active transport from hepatocytes into the bile via the bile salt export pump (BSEP) [53,57]. Conjugation increases the water solubility of BAs before their hepatic secretion [53]. Moreover, conjugated BAs are nonabsorbable and indigestible in the proximal small intestine where lipid absorption takes place [49,58]. BSEP mutations may induce the development of cholestatic liver diseases such as progressive familial intrahepatic cholestasis type 2 (PFIC-2) and benign recurrent intrahepatic cholestasis type 2 (BRIC-2). Moreover, BSEP genetic polymorphisms are associated with intrahepatic cholestasis of pregnancy (ICP) as well as drug-induced liver injury (DILI) [59]. In the intestinal lumen, BAs facilitate fat and fat-soluble vitamin uptake and undergo further modification by microbiota into secondary BAs—deoxycholic acid (DCA), lithocholic acid (LCA)—and tertiary BAs such as ursodeoxycholic acid (UDCA) [49,53]. High-fat diets stimulate BA release to the gut and result in their increased concentrations in the lumen of the large bowel with their negative impact on intestinal mucosa homeostasis and increased risk of colorectal cancer development [56,60]. BAs, present in the gut lumen, regulate hepatic BA synthesis through the farnesoid X receptor (FXR), which gives rise to the transcription of intestinal hormone fibroblast growth factor 19 (FGF19). Since FGF19 inhibits cholesterol 7α-monooxygenase in liver cells, it decreases hepatic BA synthesis (a negative-feedback loop) [61]. Moreover, the interaction of BAs with FXR causes a discharge of antimicrobial peptides (AMPs), which diminish intestinal bacterial overgrowth and accordingly prevent dysbiosis and further gut barrier dysfunction [62]. In turn, microbiota may change BA composition by favoring secondary BA production. Secondary BAs exert poorer antimicrobial effects due to their weaker FXR affinity. Therefore, intestinal BA imbalance present in the course of cholestatic liver disease may promote bacterial overgrowth [53,56,61].

Since the vast majority of conjugated BAs are reabsorbed in both the terminal ileum by the apical sodium-dependent bile acid transporter (ASBT), as well as in the colon by passive absorption and return to the liver with portal blood, more than 95% of the BA pool is preserved this way [48]. In the portal blood, conjugated bile salts binding to albumin flow back to the liver and are taken up by active transporters (sodium taurocholate co-transporting polypeptide (NTCP) and organic anion transporting polypeptide (OATP) transporter) located on the sinusoidal membrane of liver cells and then resecreted into the bile [53]. In humans, so-called enterohepatic circulation occurs about six times a day [56]. Pharmacological inhibition of the ileal apical sodium-dependent bile acid transporter (ASBT/SLC10A2) followed by a decline in intestinal BA uptake was reported to ameliorate cholestatic liver and bile duct injury in a mouse model of sclerosing cholangitis [63]. Approximately 5% of the BA pool is eliminated in feces and the small quantity of conjugated secondary BAs is absorbed in the lumen of the large bowel via passive diffusion.

### 4.2. Effects of BAs in the Human Body

Accumulating evidence reveals that BAs exert pleiotropic effects in the human body and secure various metabolic and inflammatory routes in a large number of cells, tissue types, and organs through an active interplay with host receptors and intestinal microbiota [64,65]. They not only participate in the digestion and absorption of lipids and fat-soluble vitamins but also are engaged in the feedback regulatory loop of their own hepatic synthesis [55]. BAs were reported to modify gallbladder motor function [66]. They are also involved in the gut–liver axis and related inflammatory response activation [48,52]. BAs act as signaling mediators regulating metabolic homeostasis, mainly through the nuclear farnesoid X receptor (FXR) and membrane-associated receptors such as G protein-coupled bile acid receptor 1 (GPBAR-1) and sphingosine 1 phosphate receptor 2 (S1PR2) [65,67]. Several reports indicate that BAs have an impact on epithelial cell proliferation and carcinogenesis [68,69]. They also may directly interact with gut microbiota and modify gene expression through epigenetic mechanisms [69,70,71].

As previously mentioned, BAs are produced in the liver, but they are further metabolized by intestinal microbiota. Currently, BA interactions with intestinal microbiota are increasingly recognized. Some studies indicate that the gut microbiome may influence BA uptake by modulation of ASBT action [70]. The same group of researchers reported that microbiota regulated the expression of several enzymes engaged in BA syntheses, including CYP7A1, CYP7B1, and CYP27A1. Only the 12a-hydroxylase (CYP8B1) necessary for effective cholic acid synthesis remains outside microbiota modulation [56]. It becomes clear that the gut microbiota may change the host BA pool through the complex regulations of BA synthesis and absorption. In turn, BAs may impact microbiota composition, so their interaction is bidirectional. Studies have shown that the human microbiome is shaped by BAs that support the growth of bacteria that can metabolize them and hamper the development of the other ones. Bile acids are considered powerful antimicrobials and guard the host against pathogens [71]. No wonder biliary obstruction usually results in bacterial overgrowth, intestinal barrier disruption, and bacterial translocation. The above negative effects of the disorder can be inhibited by the administration of BAs and their signaling via the farnesoid-X-receptor (FXR) [72]. Therefore, the antimicrobial impact of BAs is based not only on their direct detergent action on pathogen membranes but also on an indirect receptor-mediated release of antimicrobial factors and immune system activation [56,62]. Bile acids are agonists of so-called bile acid-activated receptors (BARs) and act as signaling molecules. Two main BARs include the farnesoid-X-receptor (FXR) and G protein-coupled bile acid receptor 1 (GPBAR1 or G-protein receptor 5), which are solely activated by BAs. The other receptors, such as pregnane X receptor (PXR), vitamin D receptor (VDR), constitutive androstane receptor (CAR), liver X receptors (LXRs), sphingosine-1-phosphate receptor 2 (S1PR2), and retinoic acid-related orphan receptor (ROR)-γt (ROR-γt), are activated by BAs together with other endogenous molecules [73]. BAs are also involved in cell signaling pathways such as c-Jun N-terminal kinase (JNK) and extracellular signal-regulated kinase (ERK) [53,74]. They can interact with cell receptors located in the gut and liver, contributing to both organs’ mutual communication and a bidirectional transfer of signals. These receptors are key players in the host’s innate immune responses. Nevertheless, nuclear FXR expression is found not only in the liver and intestine but also in other cells including those of adrenal glands, and the immune system (e.g., macrophages, the Kupffer cells, natural killer cells, and dendritic cells) [75].

However, the interplay of BAs, microbiota, and the adaptive immune response remains undefined. Recently, Song et al. reported that BA composition in the gut might be modified by both nutritional and microbial factors and have an impact on colonic FOXP3+ regulatory T (Treg) cells, which express the transcription factor RORγ [76]. The aforementioned lymphocyte subsets adjust immune response, prevent autoimmunity, and control inflammation. The researchers confirmed that restoration of the intestinal BA pool, increased colonic RORγ+ Treg counts and modulated host predisposition to colitis via BA nuclear receptors. Other reports also indicate that BAs can regulate host immune responses by modulating the Th17–Treg lymphocyte inflammatory balance [64,77]. BA gut content modifications may change BA-mediated signaling as well as the microbiome composition and induce further metabolic consequences, such as impairment of host lipid, glucose, and energy balance. Accordingly, it is strongly suggested that alterations in BA enterohepatic circulation and/or their metabolism are relevant contributors to the pathogenesis of cholestatic liver diseases, metabolic syndrome, inflammatory bowel diseases, and colorectal cancer. Therefore, BAs and their cellular receptors create interesting therapeutic targets and an important scientific field for future drug discovery.

## 5. Role of the Gut–Liver Axis in the Development of Liver Injury and Progression to End-Stage Liver Disease

Growing evidence indicates a relevant role of the gut–liver axis in the development of hepatic injury and further progression to end-stage liver disease. Seventy percent of the hepatic blood supply comes from the portal vein, which creates a direct route from the gut through the liver to the systemic circulation. Therefore, the liver remains the crucial organ of the host–gut microbiota interaction [61]. Systemic immune response corresponding to gut microbiota exposure was confirmed in humans with chronic liver diseases (i.e., alcoholic and nonalcoholic fatty liver diseases, PBC, PSC, hepatocellular carcinoma, and liver cirrhosis) [78,79,80]. Since the liver and gut are closely connected through portal circulation, they experience mutual exposure to pathogen-derived components, metabolites, and alimentary nutrients. The liver acts as a filter removing impurities and protecting the host against intestinal bacteria crossing through the gut barrier [81]. On the other hand, the liver is also capable of modifying the composition of microbiota via bile acid and immunoglobulin A (IgA) secretion [82]. Since Marshall created the theory of the gut–liver axis in 1998, growing evidence has been collected indicating the presence of interactions between the two organs [83].

### 5.1. Constituents of the Gut Barrier

The mucosal barrier of the gut relies on the mutual and accurate interaction of its multiple elements, including enterocytes connected through desmosomes, adherens junctions, and tight junctions, the secretion of antimicrobial peptides (AMPs) and IgA, and the protective capabilities of immune cells, including phagocytes and macrophages. Moreover, goblet cells produce mucin, which builds an additional protective cover on the top of the mucosal epithelium [84]. Interestingly, growing evidence indicates that besides IgA’s defensive role, it also regulates the colonization of commensal bacteria [85]. Recently, Donaldson et al. demonstrated that *Bacteroides fragilis* and other commensal species can modulate their surface structures to bind IgA, which is required for their colonization of the gut mucosal niche [86]. Taken together, IgA might modulate human susceptibility to various diseases through the regulation of commensal microbiota. The mucosal defect was suggested in PBC patients due to decreased IgA secretion from duodenal enterocytes [87].

### 5.2. Leaky Gut and its Hepatic Consequences

Accumulating evidence indicates that intestinal dysbiosis observed in the course of liver dysfunction, in particular in end-stage liver disease, enhances intestinal permeability and microbial blood penetration [87]. In turn, the leaky gut induces hepatic injury through so-called pathogen-associated molecular patterns (PAMPs) which represent components of microorganisms such as bacteria, viruses, fungi, or parasites. PAMPs cross over the intestinal wall and reach the liver with portal blood triggering alterations in immune cell activation, hepatocyte apoptosis and regeneration, and BA composition [88,89]. PAMPs present distinctive molecular attributes that differ from host cells and can be recognized via pattern recognition receptors (PRRs) that play a crucial role in the innate immune system [90]. PPRs have the potential to recognize not only PAMPs but also particles released from damaged cells called damage-associated molecular patterns (DAMPs). PRRs are classified according to their location into membrane-bound or cytoplasmic PRRs [90,91]. There are four PPR subclasses: Toll-like receptors (TLRs), nucleotide-binding oligomerization domain (NOD)-leucin rich repeat (LRR)-containing receptors (NLRs), the retinoic acid-inducible gene 1 (RIG-1)-like receptors (RLR; aka RIG-1-like helicases-RLH), and the C-type lectin receptors (CLRs) [92,93,94]. Several parenchymal and nonparenchymal hepatic cells may express PPRs. They include hepatocytes, liver sinusoid endothelial cells (LSECs), hepatic stellate cells (HSCs), Kupffer cells (KCs), and lymphocytes [95,96]. The TLRs belong to the earliest-discovered PRR subclass, and in humans consist of 10 members. Among all PPRs, TLRs have been the most extensively studied and play a critical role in the development of inflammation [93,95]. In the liver, Kupffer cells are considered the primary responders to PAMPs, although more recent studies reported TLR signaling also in hepatic nonimmune cells including hepatocytes, biliary epithelial cells, endothelial cells, and hepatic stellate cells [97,98,99]. TLRs transmit proinflammatory stimuli and induce cytokine secretion leading to liver damage, oxidative stress, and profibrogenic responses. The best-recognized cell surface TLR4 is activated by lipopolysaccharide (LPS, endotoxin), and its signaling cascade was reported as a relevant trigger of hepatic inflammatory response and fibrosis development in several types of chronic liver diseases [97,100]. Noteworthily, recently Jin et al. reported that aging-related liver degradation is associated with elevated blood LPS levels and TLR4-dependent signaling in hepatic tissue [101]. Changes in intestinal microorganism composition and related barrier dysfunction were proposed as factors involved in low-grade hepatic inflammation observed during aging and in an elderly population [102]. Endotoxins belong to well-characterized PAMPs that originate from the outer membrane of Gram-negative bacteria that cross over the impaired gut barrier. The majority of LPS comes from *Bacteroidetes* (79%). *Proteobacteria* remain only minor contributors, and *Escherichia coli* accounts for 14% of the total gut-derived LPS [103]. Of note, LPS originating from miscellaneous bacteria has been recently demonstrated to exert different and sometimes inverse effects on gut-barrier integrity, as well as the host metabolic functions, i.e., adipose tissue inflammation, glucose absorption, blood glucose concentrations, insulin, and incretin levels. LPSs’ distinctive attributes have an influence on metabolically beneficial or deleterious endotoxemia [104]. Therefore, it is strongly suggested that host metabolism is related to gut microbiota composition as well as endotoxemia levels. Under normal conditions, LPS leakage is limited by the integrity of the intestinal barrier, and only a small quantity of bacterial endotoxins can penetrate the portal bloodstream.

### 5.3. Liver Sinusoid Endothelial Cells and Kupffer Cells as the Liver Scavenging System

LPS clearance is mediated by liver sinusoid endothelial cells (LSECs), which are highly specialized regulators responsible for the first line of defense against harmful molecules derived from the digestive tract [104,105,106]. They have relevant physiological and immunological activity based on filtration, endocytosis, antigen presentation, and white blood cell recruitment [105]. The unique phenotype of hepatic sinusoidal endothelium with fenestration (open pores) and no basement membrane creates its unusual permeability, which facilitates bidirectional blood–hepatocyte communication as well as differentiates it from the capillary endothelium of other organs [105]. LSECs are exposed to preliminary contact with harmful blood molecules driving liver damage and account for the tissue response to acute or chronic injury. The response consists of the activation of hepatic cells (mainly hepatic stellate cells, the key players in liver fibrosis) and the capillarization process when the hepatic sinusoidal endothelium loses fenestrae and a basal membrane occurs [106,107,108]. The aforementioned changes are considered an initial step in hepatic fibrosis. In clinical settings, deterioration of intestinal mucosa, for example, in patients with inflammatory bowel diseases, rarely causes severe liver injury. LSECs create an important defense component in the gut–liver axis and protect the liver against colon-derived toxic constituents [109]. However, LSEC alterations together with increased LPS concentrations in the portal blood can contribute to hepatic neutrophil recruitment via the adhesion molecules intercellular adhesion molecule-1 (ICAM-1) and VCAM-1 expressed by LSECs and lead to colitis-associated hepatitis [109,110]. Most common LSEC detrimental factors include ethanol, lipid derivatives (e.g., triglycerides, free fatty acids), and virus proteins (e.g., HCV core and nonstructural protein 5A) [111,112,113,114]. All these negative stimuli may lead to endothelial cell dysfunction through the generation of oxidative stress and inflammatory response. Both LSECs and Kupffer cells create the most powerful scavenging system in the liver [105,110]. LSECs are responsible for the blood removal of soluble macromolecules and colloidal substances (i.e., particles smaller than 100 nm) by endocytosis, while Kupffer cells (KCs)—macrophages residing in the liver—are responsible for the elimination of insoluble molecules by phagocytosis [94,96,114]. Under physiological conditions, both KCs and LSECs participate in the regulation of LPS concentration in the liver and keep inflammatory responses under control. Increased gut-derived endotoxemia activates hepatic KCs and initiates their profibrogenic and proinflammatory effects [97,115]. In the bloodstream, LPS binds to the lipopolysaccharide-binding protein (LBP) and the complex reaches the liver. Kupffer cells are reported to intercept 90 percent of free LPS (aggregates, bacterial membrane fragments, or loosely bound to albumin, CD14, or other proteins) that penetrate the portal circulation [116]. KC activation occurs through the interaction of the LPS–LBP complex with cell membrane receptors: surface CD14 and transmembrane TLR4. The CD14 protein has a high affinity for endotoxin and mediates TLR4 recognition of LPS. CD14 connects to LPS and transfers it to the TLR4–myeloid differentiation factor 2 (MD2) complex [61,100,117]. TLR4–MD-2 complex aggregation triggers the activation of multiple signaling mediators, such as nuclear factor-kappa B (NF-κB) and interferon regulatory factor 3 (IRF3). As a result, the intensive synthesis of oxygen free radicals, chemokines, and proinflammatory cytokines including Tumor Necrosis Factor-alpha (TNF alpha), IL-1, IL-6, IL-12, and IL-18 occur [118,119]. The inflammatory cascade generates subsequent recruitment, activation, and accumulation of peripheral immune cells in the liver [88]. As mentioned previously, TLR4 can also be activated by endogenous and host-derived particles called DAMPs (e.g., mitochondrial DNA) released by uninfected injured tissue or dying cells causing sterile inflammation [95,120]. The process may be important for tissue repair/regeneration, but may also induce the development of different inflammatory or autoimmune diseases and cancer [120,121]. The aforementioned inflammatory cascade triggered through gut–liver axis impairment and related to intestinal dysbiosis may contribute to persistent liver inflammation, subsequent fibrosis, disease progression, conversion to cirrhosis, and end-stage liver failure.

## 6. Dysbiosis in Immune-Related Cholangiopathies

There are 10^13^–10^14^ total microbial cells in the human intestinal tract, with more than 1000 bacterial species [122,123,124]. Human gut flora consists of bacteria, fungi, protozoa, viruses, and archaea [84]. Two major phyla include *Bacteroidetes* and *Firmicutes. Bacteroides*, *Eubacterium*, *Bifidobacterium*, *Ruminococcus*, *Peptostreptococcus*, *Propionibacterium*, *Clostridium*, *Lactobacillus*, *Escherichia*, *Streptococcus*, and archaeal genus *Methanobrevibacter* are prevalent genera. Phyla such as *Proteobacteria*, *Verrucomicrobia*, *Actinobacteria*, *Fusobacteria*, and *Cyanobacteria* are present in relatively smaller proportions [125,126]. The human microbiome composition plays a critical role in intestinal homeostasis and depends on early and later life circumstances, such as individual genetics, mode of birth, breastfeeding, dietary habits, lifestyle, stress, aging, and medical treatment [127,128]. Altered microorganism composition, reduction of their diversity and stability, as well as an increased number of proinflammatory bacteria, are typical features of gut dysbiosis. The involvement of microbiota dysbiosis in human diseases remains under deep investigation [84,129]. Growing evidence indicates that transformations in the gut microbiota may also induce liver injury. The possible association of BAs, microbiome deviations, and cholestasis have been actively investigated recently and seem to be implicated in the etiopathogenesis of PBC and PSC [123,130,131,132,133,134,135,136]. Since up to 75% of PSC patients present with accompanying IBD, PSC may be regarded as a typical example of the gut–liver interconnection [137]. Enhanced gut immune reactions, as well as abnormal intestinal permeability with increased blood concentrations of microbial products, have been reported in patients with PSC and PBC in comparison with healthy individuals [138,139,140]. Moreover, examination of liver specimens from PSC and PBC patients revealed abnormal accumulation of LPS in biliary epithelial cells [141]. The aforementioned results strongly suggest the possible contribution of intestinal microbiota to PSC and PBC etiopathogenesis. The vicious circle of gut–liver crosstalk in the course of immune-related cholangiopathies is summarized in Figure 1.

Generally, intestinal dysbiosis can result in two different outcomes: an imbalance between protective and detrimental metabolites, and/or an altered immune response with the recruitment of activated T cells, as well as chemokine and integrin receptor overexpression, that break immune homeostasis and facilitate harmful effects in the liver. Progression of cholestatic liver disease ruins the normal gut microbiota, which in turn aggravates bile duct dysfunction, creating a vicious circle [123]. Several prior studies have confirmed considerable differences in the gut microbiome of PSC and PBC patients in comparison with healthy individuals [130,131,132,142]. Growing evidence indicates that altered microbiome composition can affect the course of cholestatic liver disease via different mechanisms. Recently, Kummen et al. proposed the categorization of complex host–microbiome interplay into three biomes i.e., the endobiome, immunobiome and xenobiome [143]. The endobiome is associated with the bacterial synthesis of endogenous molecules relevant to immune and metabolic host status, such as secondary BAs (lithocholic acid, ursodeoxycholic acid), branched-chain fatty acids, indoles, phenols, ammonia, amines, and vitamins K and B. The immunobiome includes the generation of specific microbial components that trigger host immune responses. They consist of PAMPs (e.g., LPS), but also peptide and lipid antigens that are presented to various immune cells. The xenobiome relates to the bacterial genome encoding products (e.g., carbohydrate-degrading enzymes) allowing the processing of chemical substances (e.g., medications) or nutritional ingredients. For example, it facilitates the fermentation of carbohydrates via colonic microbiota with subsequent generation of nutrients important for the integrity of intestinal epithelium, e.g., short-chain fatty acids (SCFAs) [143]. Recent data obtained from human and animal models indicate that intestinal microorganisms may operate as PSC and PBC course modifiers. The underlying pathogenetic mechanisms by which they act in both cholangiopathies are not fully understood, but ongoing evidence indicates that bile acid imbalance, leaky gut with microbial translocation, and some obscure (so far) immune alterations should be taken into account.

### 6.1. Gut Microbiome Alterations in Patients with PSC

A recent study of two independent case–control cohorts from Norway and Germany performed with state-of-the-art sequencing methods identified huge functional differences in the gut microbiome of patients with PSC [144]. PSC patients presented with numerous alterations of gut microbiome, including fewer microbial genes, an increased prevalence of *Clostridium* species, but decreased *Eubacterium* spp. and *Ruminococcus obeum,* marked impairment of genes related to vitamin B_6_ and branched-chain amino acid synthesis, with further alterations in blood concentrations of related gene products and corresponding decreased liver transplantation-free survival. Previous studies revealed that *Veillonella* stool content was higher in PSC patients than in healthy individuals [131]. *Enterococcus, Streptococcus, Lactobacillus*, and Proteobacteria such as *Escherichia coli* were also frequently enriched [132,145]. However, a relative reduction in the number of *Firmicutes,* such as *Faecalibacterium prausnitzii* and *Ruminococcus gnavus*, bacteria that produce butyrate, was reported [131,132,134,144]. A recent study by Vieira-Silva et al. revealed that in 106 patients with PSC and/or IBD, *Fusobacterium* was related to intestinal inflammation, while *Enterococcus* was related to cholangitis/biliary obstruction [145]. Moreover, Rühlemann et al. reported that fecal bacterial composition could identify PSC with AUC 0.88 [146]. Several studies indicate that there are no significant microbiome differences between patients with PSC with or without concomitant IBD [132,134,146]. However, the microbial profile in PSC is different in comparison with healthy individuals as well as IBD patients without PSC [131].

### 6.2. Gut Microbiome Alterations in Patients with PBC

Growing evidence indicates a relevant role of gut microbiota in PBC pathogenesis [147,148,149]. As reported, PBC may influence the gut microbiome composition through alterations of intestinal motility, immune response, bile acid secretion, as well as portal hypertension development. Antimitochondrial antibodies present in 90–95% of PBC patients, which can occur years before the disease’s onset, react to the pyruvate dehydrogenase complex E2 (PDC-E2) expressed by the biliary epithelium [150,151]. Several reports showed that the synthesis of antibodies against bacterial proteins as well as the molecular mimicry of microbial proteins and PDC-E2 are critical for PBC development [152,153,154,155]. Observations of the association of PBC with recurrent urinary infections caused by *Escherichia coli* support this suggestion, especially in females [156,157]. Moreover, long-term microbial exposure in an animal model also induced autoantibody secretion and PBC resembling histology of the liver [158]. There is growing evidence that gut microbiota alterations might be directly related to PBC development by their interplay with host metabolism and immunity. Lv et al. demonstrated that in PBC patients, some potentially beneficial bacteria, such as *Acidobacteria, Lachnobacterium* spp., *Bacteroides eggerthii* and *Ruminococcus bromii*, were depleted and other bacterial taxa containing opportunistic pathogens, such as *γ-Proteobacteria, Enterobacteriaceae, Neisseriaceae, Spirochaetaceae, Veillonella, Streptococcus, Klebsiella, Actinobacillus pleuropneumoniae, Anaeroglobus geminatus, Enterobacter asburiae, Haemophilus parainfluenzae, Megasphaera micronuciformis,* and *Paraprevotella clara*, were enriched [130]. Results of recent studies strongly suggest that the composition of gut microorganisms could create a specific disease bioindicator and serve as a biomarker for future diagnostic approaches or as a new therapeutic target. Tang et al. described gut microbial profiles in patients with PBC [135]. They reported significantly reduced bacterial abundance in PBC compared with healthy controls and showed that the microbiota profile presented good accuracy (AUC 0.84) for PBC patient identification. Their study revealed a significant reduction of *Bacteroidetes* spp., *Suterella, Oscillospira,* and *Faecalibacterium,* as well as a significant increase in *Haemophilus, Veillonella, Clostridium, Lactobacillus, Streptococcus, Pseudomonas, Klebsiella.* Moreover, dysbiosis in the PBC cohort was partially reversed by UDCA therapy. These observations may suggest that conversion of microbiome composition is possible based on the bacteria’s bile acid sensitivity and/or their involvement in the bile acid metabolism and are consistent with previous reports regarding microbiota–bile acid interplay [70,159]. Taken together, altered intestinal microbiota may impact gut motility, immune defense, bile secretion, and patient response to UDCA treatment and lead to poor PBC outcomes. Considering the aforementioned results, bile acid profiling could contribute to PBC patients’ diagnosis and disease status assessment.

## 7. Current Treatments in PBC and PSC

As mentioned previously, despite large-scale research and due to still poorly explained pathogenesis of both immune-related cholangiopathies, therapeutic options in PBC and PSC are limited and not satisfactory [3,4,5,160]. Notably, in contrast to PBC, PSC’s natural course is difficult to define and in order to assess its treatment efficacy, clinical trial design, as well as primary endpoints, should be determined and clarified. No pharmacological treatment has been proven successful to improve long-term prognosis in PSC patients so far [161]. Currently, several new pharmacological treatments are under investigation, representing potential to improve PBC and PSC clinical outcomes [162]. Medications for the therapy of patients with PBC and PSC, which are available now and promising new agents for the near future, are presented below.

### 7.1. Ursodeoxycholic Acid (UDCA) and Norursodeoxycholic Acid (norUDCA)

Since the early 1970s, chenodeoxycholic acid (CDCA), a primary bile acid, and ursodeoxycholic acid (UDCA), a secondary bile acid, have been shown to be effective in promoting the dissolution of cholesterol gallstones [73]. Currently, UDCA is recommended by the European Association for the Study of the Liver (EASL) [19] and the American Association for the Study of Liver Diseases (AASLD) [8] as the first-line therapy in PBC, as it slows the progression of liver disease [163,164]. It delays histological progression and extends the transplant-free survival rates among PBC patients [165,166]. About 90% of a therapeutic dose of UDCA is absorbed in the small bowel after oral administration. After intestinal absorption, UDCA enters the portal vein and undergoes efficient extraction from portal blood by the liver, where it is conjugated with either glycine or taurine [167]. Several UDCA modes of action have been confirmed in the course of cholestatic liver diseases. UDCA can diminish intrahepatic biliary epithelial cell injury through several mechanisms that include modification of the bile acid pool, but also by its choleretic, cytoprotective, antiapoptotic, antioxidant, and probable immunomodulatory action [168,169,170]. UDCA administration leads to reduced cholesterol absorption and higher bile acid synthesis. Since it supports cholesterol transformation to bile acids, UDCA decreases cholesterol content in the bile. In contrast to other bile acids, UDCA is noncytotoxic since it has less affinity to cell membranes and does not solubilize them [171]. Moreover, UDCA stimulates biliary HCO3− secretion in animal models and PBC patients, so it may restore the protective bicarbonate (HCO3-) umbrella in the biliary epithelium [172]. Notably, as reported by Adamowicz et al., UDCA may prevent the suppression of peroxisome proliferator-activated receptor alpha (PPARα), which has an important role in both the inhibition of excessive inflammatory responses and cell apoptosis [173]. Furthermore, a recent meta-analysis suggests that UDCA treatment might be associated with a significant decrease in total cholesterol in PBC patients [174], but also exerts a beneficial effect on glucose homeostasis, as it significantly reduces fasting blood glucose and HbA1c levels, as well as serum insulin concentrations [175]. Moreover, UDCA enhances NF erythroid 2-related factor 2 (NFE2L2) expression and UDCA-induced suppression of IFN-γ and C-X3-C Motif Chemokine Ligand 1 (CX3CL1) production attenuates the chemotactic and adhesive abilities of liver-infiltrating T cells in PBC [176]. With the above mechanisms, UDCA can reduce cholangiopathy progression. However, for PSC, UDCA treatment is still controversial because it does not improve survival rates [6]. According to Zhu et al., in patients with PSC, metronidazole plus UDCA treatment was the most effective therapy in patient survival rates and impact on histological disease progression [177]. In patients with PBC, oral UDCA is used at dosages of 13–15 mg/kg/day. If tolerated, treatment should usually be lifelong [9]. Experts from the British Society of Gastroenterology suggest that in UDCA-treated PBC patients with an ALP above 1.67 × the upper limit of normal (ULN) range and/or bilirubin above 2 × ULN, the addition of second-line therapy should be considered [9]. Ten-year survival among UDCA-treated PBC patients is slightly lower than in the general population [178]. This requires searching for novel therapies that would increase the survival rates among PBC patients. As mentioned before, UDCA efficacy is limited in patients with PSC. Since it improves biochemical hepatic parameters, it is commonly administered in PSC cases [179,180]. However, no favorable UDCA impact has been confirmed in long-term PSC outcomes.

An artificial homologue of UDCA i.e., norursodeoxycholic acid (norUDCA), has been introduced recently as a novel therapeutic approach in cholestatic and metabolic liver diseases with a specific focus on PSC treatment [181]. Compared to UDCA, norUDCA presents relative resistance to taurine and glycine conjugation, which enables its cholehepatic shunting. As a result, bicarbonate ion and bile acid secretion increases, which protects the bile tree and the liver. Results obtained from a recent clinical trial revealed that norUDCA significantly reduced serum ALP levels in a dose-dependent manner and showed a favorable safety profile comparable to placebo [182]. Noteworthily, a direct effect of norUDCA on CD8+ T cells with a decrease in excessive CD8+ T cell-driven hepatic inflammation was observed in a murine model of cholestasis, which is relevant for the treatment of immune-mediated cholangiopathies such as PSC [183]. Treatment with norUDCA in PSC patients seems promising and should be further evaluated in multicenter clinical studies.

### 7.2. Obeticholic Acid (OCA)

Obeticholic acid (OCA, 6-ECDCA, INT-747), a semisynthetic hydrophobic bile acid analogue, is the first-in-class highly selective agonist of farnesoid X receptor (FXR) [184]. As the FXR agonist, OCA presents 100 times higher potency in comparison with endogenous BAs [185]. OCA, by activating FXR, stimulates intrinsic pathways leading to decreasing hepatic BA synthesis and uptake and increasing BA outflow from the liver. In detail, upon activation, FXR binds to the retinoid X receptor (RXR) and finally causes the transcriptional inhibition of rate-limiting enzymes in BA synthesis, i.e., cytochrome P450 (CYP)7A1 and liver receptor homologue 1 (LRH-1) [186]. Moreover, FXR inhibits the sodium taurocholate cotransporting polypeptide (NTCP) and decreases hepatic BA uptake [187]. Increased BA outflow from the liver to the bile duct lumen results from FXR agonist induction of the transporter bile salt export pump (BSEP) and multidrug-resistant protein-3 (MDR3) [188], but also from increased expression of the organic solute transporters OSTα and β [189]. A recent study by Kjærgaard et al., who used a special scanning technique (PET scan), showed that OCA increased the transport of bile acids from the blood into the bile. Consequently, OCA reduced the time of hepatocyte exposure to cytotoxic BAs [190]. Noteworthy, in vitro, OCA inhibited both LSEC and Kupffer cell activation and reduced their proinflammatory cytokine secretion, resulting in diminished hepatic stellate cell activation. As a result of OCA administration, anti-inflammatory and antifibrotic effects can be seen in the liver [191].

OCA is the only registered agent for second-line treatment in PBC patients UDCA-intolerant and/or UDCA nonresponders for whom a 12 month-treatment has not produced any benefit [192]. It has been examined in PBC patients with inadequate response to UDCA and has shown promising results [165]. OCA is the only therapy licensed by the US Food and Drug Administration (FDA) and the European Medicines Agency (EMA) and endorsed by the National Institute for Health and Care Excellence (NICE) as second-line therapy used in combination with UDCA for PBC patients who have an inadequate response to at least 1 year of UDCA treatment, or as monotherapy for those who are intolerant to UDCA [184]. Kjærgaard et al. showed that OCA increased the transport of bile acids from blood to bile and reduced the time that potentially toxic bile acids reside in the liver by approximately a third [190]. FXR modulates the expression of pathway-specific as well as polyspecific transporters and enzymes, thereby acting at the interface of BA, lipid, and drug metabolism. OCA was also tested in patients with type 2 diabetes and nonalcoholic fatty liver disease (NAFLD) as it increased insulin sensitivity, and reduced markers of liver inflammation and fibrosis in patients with type 2 diabetes mellitus and NAFLD [193]. The recommended starting dose is 5 mg by mouth daily; however, patients may uptitrate to 10 mg daily after six months to improve response [194]. Pruritus is the most frequent adverse event in OCA-treated patients and can lead to discontinuation in almost 10% of cases [184,195]. Additionally, alterations in serum lipid levels may show up. Among them, the most common is a small decrease in high-density lipoprotein cholesterol [184]. Furthermore, the high cost of OCA may limit its use in some health systems in the world. Soret et al. used triple therapy that contained UDCA, OCA, and bezafibrate or fenofibrate in PBC patients after failure to second-line therapy. After a mean duration of 11 months of triple treatment, they observed normalization of ALP, GGT, ALT, AST, and bilirubin levels and improved pruritus in the study group [196].

The efficacy and safety of OCA have also been assessed in PSC patients. Results of a randomized, placebo-controlled, phase II study confirmed that it reduced serum ALP during an initial 24-week treatment period and further on during the 2-year, long-term extension of the study [197].

### 7.3. Regulators of Bile Acid Homeostasis

Unfortunately, about 40% of all PBC patients fail to respond to UDCA therapy and remain at high risk of further complications. Under such circumstances, it is essential to start second-line treatment in this PBC subgroup, such as fibrates or obeticholic acid as UDCA added therapy [198]. Patients with advanced PBC stages (i.e., III or IV) do not respond adequately to UDCA and are candidates for supplementary treatment [199]. Recognition of the moment to stop UDCA monotherapy is crucial in the course of PBC. There are special criteria that should be checked in symptomatic PBC patients every six months including Paris-2, Toronto, GLOBE, and so forth. PBC patients presenting with high levels of total and conjugated bilirubin and/or AP more than 1.5-fold the upper limit of normal (ULN) should be considered candidates for second-line therapy [200].

#### 7.3.1. Peroxisome Proliferator-Activated Receptor (PPAR) Agonists

Fibrates are antilipemic agents that lower cholesterol and triglyceride levels. They act as agonists of peroxisome proliferator-activated receptors, and in combination with UDCA have shown potential benefit in patients with PBC [201,202]. Bezafibrate was first reported as biologically effective in patients with PBC who were refractory to UDCA in 1999 [23]. Chung et al. showed that in PBC patients refractory to UDCA, administration of complex treatment with fibrates added to UDCA leads to a higher probability of ALP normalization and decreases the risk of liver cirrhosis development in comparison with UDCA monotherapy [203]. Pareset et al. also used fibrate as an added therapy to UDCA in PBC patients and observed normalization of ALP in half of the study subjects. Furthermore, their research showed major improvement in pruritus [204]. The study by Wang et al. analyzed UDCA-refractory PBC patients with a subgroup receiving combined therapy with UDCA and fenofibrate. Although no significant differences in the final histological evaluation were found after 3 years of the aforementioned treatment, patients who received both drugs presented with stabilization of fibrosis (85.7%), and bile duct loss (78.6%) compared to the group treated with UDCA alone (70.6% and 76.5%, respectively) [205]. Tanaka et al. reported that bezafibrate administered to UDCA-refractory patients caused normalization of ALT levels and decreased the rate of symptom occurrence in asymptomatic PBC patients [206]. Honda et al. confirmed the above data and showed that the long-term prognosis was better in UDCA-refractory patients when bezafibrate was added, particularly in early-stage PBC patients [207]. Seladelpar is a potent, selective agonist PPAR-δ, which is implicated in bile acid homeostasis. In a randomized phase II study, seladelpar in doses of 50 mg/day and 200 mg/day normalized ALP activity in PBC patients who completed 12 weeks of treatment. However, treatment was complicated by a significant increase in ALT and AST levels and stopped early [208]. Kremer et al. continued the research with seladelpar for 1 year and concluded that the PPAR agonist led to consistent improvement in both symptom burden and biochemical response [209]. Bowlus et al. observed that seladelpar caused ALP normalization as well as an improvement in the pruritus visual analogue scale score [210]. Elafibranor, a dual PPARα/δ agonist, was investigated in patients with PBC and had significantly reduced levels of ALP and GGT. Pruritus was not induced or exacerbated by elafibranor. All observed and possibly drug-related adverse events were mild to moderate [211].

#### 7.3.2. Apical Sodium-Dependent Transporter (ASBT) Inhibitors

As mentioned before, the apical sodium-dependent transporter (ASBT) protein, which is located in the terminal ileum, plays an essential role in bile acid homeostasis. Alterations in bile acid circulation occur in the course of PBC and PSC and contribute to the development of pruritus, which can be modified by ASBT inhibitors [212]. ABST inhibitors are also called ileal bile acid transporter (IBAT) inhibitors [213]. An experimental study showed that inhibition of intestinal bile acid absorption by ASBT inhibitor A4250 attenuates bile acid-mediated cholestatic liver injury by reducing bile acid output [214]. Furthermore, after treatment with the ASBT inhibitor linerixibat, which reduces pruritus scores in PBC patients, fecal bacterial composition significantly changed from baseline. Notably, these changes might be due to the increased bile salt load in the colon and resulting from ASBT inhibition [215,216]. In a study by Hegade et al. GSK2330672, a selective inhibitor of human ileal bile acid transporter (IBAT) was investigated in patients with PBC. In PBC patients who presented with pruritus, 14 days of treatment with GSK2330672 was generally well tolerated with no serious adverse events and demonstrated efficacy in reducing pruritus severity. Diarrhea was the most frequent adverse event during this treatment [212]. In patients with PSC, drugs targeting the gut–liver axis such as vedolizumab appear to be promising based on the close link between PSC and IBD [217]. Nowadays, ASBT inhibitors and rituximab are still under close evaluation for the treatment of pruritus and fatigue [218]. Unfortunately, the study by Khanna et al. reported that rituximab, although safe over the 12-month study period, showed no evidence of effectiveness for the treatment of fatigue in PBC [219].

#### 7.3.3. Farnesoid X Receptor (FXR) Agonists

The next group of medications that have been reported to exert promising effects and reduce liver damage in patients with nonalcoholic steatohepatitis (NASH) and PBC includes FXR agonists. Several new FXR agonists are currently under assessment, for example, cilofexor, and tropifexor [220]. Tropifexor was investigated in an animal model of liver injury in rats with obstructive jaundice. The tropifexor animal group revealed statistically significant decreases in the values of GGT, total bilirubin, and direct bilirubin. In humans, a phase II, double-blind, placebo-controlled study with tropifexor showed decreases in liver enzymes, i.e., GGT, ALP, and ALT at or before the 28th day of treatment. Pruritus was the most frequent adverse event (AE) in study groups [221]. Cilofexor, an oral, potent, and selective nonsteroidal FXR agonist has demonstrated anti-inflammatory and antifibrotic effects and good results in decreasing portal pressure in preclinical models of liver fibrosis. In the next study, cilofexor 30 mg or 100 mg per day administered for 12 weeks induced significant declines in serum ALP, ALT, AST, and GGT. Furthermore, cilofexor did not exacerbate pruritus in PSC patients [222].

#### 7.3.4. Other Therapeutic Options

Budesonide, a corticosteroid with an extensive first-pass hepatic metabolism, appeared promising for the treatment of PBC [223]. In a study by Hirschfield et al., patients with PBC who received budesonide presented with a decrease in serum ALP, and the percentage of patients with normal bilirubin levels was significantly higher in the budesonide than in the placebo group. In contrast to placebo, budesonide reduced mean ALP values and up to 35% of budesonide-treated patients achieved ALP normalization [224]. Angulo et al. reported that there was an improvement in serum levels of total bilirubin, but only a marginal change in serum ALP in PBC patients treated with UDCA and 9 mg of oral budesonide daily for 1 year [223].

Baricitinib, an orally administered inhibitor of Janus kinase 1 and 2, was assessed in PBC patients by Gordon et al. In the randomized, double-blinded, placebo-controlled 12-week trial, the baricitinib-treated patient demonstrated a 30% decrease in serum ALP. A single nonserious treatment-emergent adverse event of moderate sinusitis was reported by the baricitinib-treated patient on day 47 [225].

Other reports indicated that PSC patients PSC treated with vancomycin presented with significant reductions in their serum ALP and bilirubin levels, and improvements in fatigue and pruritus were also observed [226,227,228]. Ali et al. showed that oral vancomycin administered at a dose of 500 mg three times daily in PSC patients caused a reduction in GGT, ALP, and ALT within 6 months. Vancomycin therapy in PSC was well tolerated [229]. Emerging treatments in PBC and PSC patients are presented in Table 1.

### 7.4. Probiotics, Prebiotics, Postbiotics, and Synbiotics in PBC and PSC

Gut microbiota can be modified by the supplementation of probiotics, prebiotics, postbiotics, and synbiotics. According to the 2002 World Health Organization (WHO) definition, probiotics are live microorganisms administered in appropriate amounts, which have a positive effect on host health [230]. Prebiotics are traditionally indigestible food ingredients that selectively stimulate the growth and activity of a limited number of bacteria in the digestive tract [231]. Nowadays, we are observing a growing interest in postbiotics. Although postbiotics do not contain live microorganisms, they show a beneficial health effect similar to probiotics with no risks associated with their intake. Synbiotics are a combination of prebiotics and probiotics that are claimed to have a beneficial impact on the gut microbiome. Postbiotics play a vital role in the maturation of the immune system. As such, they may be useful in treating various diseases, e.g., inflammatory bowel diseases [230]. Furthermore, dysbiosis as previously mentioned may play a critical role in the pathogenesis of immune-related liver diseases [232]. Disease-specific microbiome alterations were observed in patients with AIH, PBC, PSC, and IBD. Changes in microbiome composition in patients with PBC and PSC are summarized in Table 2.

There is scarce evidence of the potential beneficial effects of probiotic administration in PBC and PSC so far. Results of a randomized controlled trial conducted by Vleggaar et al. [233] showed that 3 months of treatment with four *Lactobacillus* and two *Bifidobacillus* strains had no beneficial effects on symptoms, liver biochemistry, or liver function among patients with PSC. The next report by Shimizu et al. [234] presented a single case of a 13-year-old boy with PSC and undetermined colitis who was successfully treated with a combination of a steroid (prednisolone, 30 mg/day), salazosulfapyridine (3000 mg/day), and a probiotic (*Lactobacillus casei Shirota*, 3 g/day). Notably, recent research confirmed hepatoprotective properties of *Lactobacillus plantarum Lp2*, which ameliorated liver injury by inhibiting LPS-induced hepatic inflammation, decreasing hepatocyte oxidative damage and apoptosis and mitigated cyclophosphamide-induced liver damage by inhibiting mitochondrial-mediated hepatocyte apoptosis [235,236]. However, further multicenter studies are required to confirm existing knowledge and objectively determine probiotics’ efficacy as an adjunctive treatment in PBC and PSC patients. Currently, there are only two clinical studies registered on https://clinicaltrials.gov/ for probiotic investigation in immune-related cholangiopathies. They are presented in Table 3.

### 7.5. Fecal Microbiota Transplantation (FMT) in Immune-Related Cholangiopathies

Over the past decade, fecal microbiota transplantation (FMT) has gained growing attention due to its efficacy in gut microflora reconstruction, especially in patients with recurrent Clostridium difficile infection (CDI) [237,238]. Moreover, FMT has been reported as a relatively safe, well-tolerated, and successful treatment for various disorders other than CDI, with evidence in active UC being the most compelling [239]. FMT can be performed via the upper or lower gastrointestinal (GI) tract to restore normal intestinal homeostasis [240]. So far, only one clinical trial and one case report have described FMT performance in immune-related cholangiopathies, specifically in patients with PSC. In 2018, the first case report by Philips et al. [241] presented a 38-year-old male diagnosed with PSC and no IBD who developed recurrent episodes of bacterial cholangitis (BC) within 6 months. The patient was listed for liver transplantation (LT) and then the next BC episode occurred. Four sessions of FMT were performed. As a result, BC was ameliorated and the patient remained afebrile and unicentric for 1 year [241]. In a recent pilot study by Allegretti et al. [242], 10 PSC patients with concurrent IBD underwent a single FMT by colonoscopy. Nine patients had UC, and one had Crohn’s colitis. Patient baseline ALP values were greater than 1.5-fold the upper limit of normal (ULN). After the FMT treatment, 30% of PSC patients experienced ≥50% decrease in ALP levels. Moreover, post-FMT, within the first week, a stable increase in microbial diversity and donor microbiota engraftment were observed [242]. Targeting the intestinal microbiome gives rise to great scientific enthusiasm and constitutes a promising approach to the medical management of the liver as well as other dysbiosis-related diseases. However, it needs additional exploration in preclinical and clinical controlled trials.

## 8. Conclusions

PSC and PBC are progressive immune-related cholangiopathies with no precisely defined pathogenesis in which cholangiocytes form the main target of hepatic damage. The mechanisms triggering an inflammatory response, bile duct destruction, alterations in bile flow, and subsequent hepatic fibrosis, remain poorly understood and await further complex investigations. Even though the prevalence of the aforementioned cholangiopathies is not high, both disorders, particularly in their advanced stages, remain a frequent indication for liver transplantation. However, such a therapy may not be a definite one, as about 30–40% of patients undergoing liver transplantation present a recurrence of their original illness [2]. Data obtained from clinical research conducted on patients with PBC and/or PSC as well as on animal models of cholestasis highlight the key role of immune cells in the pathogenesis of both cholestatic disorders. Nevertheless, currently available treatments directed towards bile acid toxicity (i.e., UDCA and norUDCA) or bile acid receptors and their signaling routes (i.e., FXR agonists, agonists of TGR5 and PPAR, fibrates) are not sufficiently effective and do not fulfill all medical expectations or patient needs. Recent growing evidence indicates that gut dysbiosis may exert a crucial role in intestinal wall permeability and mucosal barrier devastation. Therefore, it may be a relevant contributor to microbial as well as other molecule penetration through the portal blood into liver parenchyma and further negative hepatic consequences. Accordingly, shaping the gut microbiome could be an important therapeutic direction, as demonstrated in preclinical and clinical models. Considering the aforementioned study results, treatments focusing on specific microbiota tailoring might constitute promising future therapeutic strategies in PBC and PSC medical management.

## Figures and Tables

**Table 1:** Emerging therapies in PBC and PSC *.

Mechanism of Action	Agent	Main Results	Most Common Side Effects
Agonists of peroxisome proliferator-activated receptors (PPAR)	Bezafibrate [203,204,206,207]	normalization of ALP and ALT improvement in pruritus improvement in long-term prognosis	muscle pain renal dysfunction
	Fenofibrate [203,205]	stabilization in fibrosis stabilization in bile duct loss	
	Seladelpar [209,210]	improvement in pruritus normalization of ALP	diarrhea nausea
	Elafibranor [211,212]	decrease of ALT, ALP, and GGT	increase of creatinine
ASBT inhibitors	Linerixibat [215,216]	improvement in pruritus	diarrhea abdominal pain
Agonists of farnesoid X receptor (FXR)	Cilofexor [222]	decrease of ALT, AST, ALP, and GGT	pruritus
	Tropifexor [221]	decrease of ALT, ALP, and GGT	
Corticosteroids	Budesonide [223,224]	normalization/decrease of ALP decrease of bilirubin	headache nasopharyngitis pruritus
Janus kinase 1 and 2 inhibitors	Barticinib [225]	decrease of ALP	moderate sinusitis
Antibiotics	Vancomycin [228,229]	decrease of ALT, ALP, and GGT normalization of bilirubin improvement in pruritus	rare

* ALP (alkaline phosphatase), ALT (alanine transaminase), ASBT (apical sodium-dependent bile acid transporter), AST (aspartate transaminase), GGT (gamma-glutamyltransferase).

**Table 2:** Changes in gut and oral microbiome profiles in PBC and PSC.

Microbiota Profile	Bacterial Genera in PBC Patients	Bacterial Genera in PSC Patients
enriched ↑	Proteobacteria [130,132,145] Veillonella [130,131,135] Lactobacillus [132,135,145]	Proteobacteria [130,132,145] Veillonella [130,131,135] Lactobacillus [132,135,145]
	Haemophilus [130,135] Clostridiales [131,132,135] Streptococcus [130,132,135,145] Pseudomonas [135] Klebsiella [130,135] Enterobacteriaceae [130] Neisseria [130]	Enterococcus [132,145] Bacteroidetes [130,135]
depleted ↓	Firmicutes [132,145] Bacteroidetes [130,135]	Firmicutes [132,145] Bacteroidetes [130,135]
	Sutterella [135] Oscillospira [135] Faecalibacteria [135]	

**Table 3:** Registered clinical trials of probiotic investigation in immune-related cholangiopathies (accessed through the website https://clinicaltrials.gov/ on 7 January 2023).

ClinicalTrials.gov Identifier/Location	Disease	Intervention	Study Design	Status	Primary Outcome Measures	Secondary Outcome Measures
NCT03521297 China	PBC patients with poor UDCA response	Probiotics (Micro V Probiotics)	Randomized, placebo-controlled, interventional; Phase 2	Not yet recruiting	percentage of patients with the biochemical response (serum ALP or GGT decreased by 20% from baseline)	
NCT00161148 Netherlands	PSC patients with IBD	Probiotics (not defined)	Double-blind randomized cross-over pilot study; phase 3	Unknown	probiotics’ effect on serum liver tests	probiotics’ effect on fatigue and pruritus

## Data Availability

All data are included in the main text.
